# Combining Advanced Targeted Therapy in Inflammatory Bowel Disease: Current Practice and Future Directions

**DOI:** 10.3390/jcm14020590

**Published:** 2025-01-17

**Authors:** Alice De Bernardi, Cristina Bezzio, Michele Puricelli, Daniela Gilardi, Simone Saibeni

**Affiliations:** 1 IBD Unit, Gastroenterology Unit, Rho Hospital, ASST Rhodense, 20017 Rho, Italy; aldebernardi@asst-rhodense.it (A.D.B.); michele.puricelli03@universitadipavia.it (M.P.); dgilardi@asst-rhodense.it (D.G.); 2 IBD Centre, IRCCS Humanitas, Research Hospital, 20089 Rozzano, Italy; cristina.bezzio@hunimed.eu; 3 Department of Biomedical Sciences, Humanitas University, 20072 Milan, Italy

**Keywords:** ulcerative colitis, Crohn’s disease, inflammatory bowel disease, therapy, biologics, small molecules, Dual Targeted Therapy, combined advanced therapy

## Abstract

**Background/Objectives:** Despite the increasing number of available medications, a significant proportion of IBD patients fail to achieve the current therapeutic targets. Uncontrolled IBD has a significant impact on patients’ quality of life and on overall costs for the healthcare system. Given the complex pathophysiology of IBD, Combined Advanced Targeted Therapy (CATT), involving the combination of biologics/small molecules, appears to have biological plausibility and is gaining increasing interest. The aim of this narrative review is to provide the current evidence regarding CATT in IBD and propose future developments in this field. **Methods:** Relevant literature evidence was searched with pertinent MeSH terms in the most important database. **Results**: Available evidence of CATT in IBD provides encouraging results in terms of efficacy and effectiveness, with an acceptable safety profile. CATT may represent a therapeutic solution for patients with “difficult-to-treat” IBD or with concomitant immune-mediated inflammatory diseases. However, current data are restricted by an overall low level of evidence and by the short follow-up. **Conclusions:** There are no data concluding the superiority of one combination therapy over another. Various therapeutic schemes could be applied in the near future. Further studies are needed to provide recommendations and integrate this therapeutic strategy into everyday clinical practice.

## 1. Introduction

Inflammatory bowel diseases (IBDs) are a group of chronic inflammatory disorders that primarily affect the digestive system, with Crohn’s disease (CD) and ulcerative colitis (UC) being their major subtypes [1,2].

The natural history of these conditions is characterized by a chronic course, with alternating periods of flares and remission. During active phases, IBD patients may experience typical intestinal symptoms (diarrhea, hematochezia, abdominal pain, fecal urgency) and, in the most severe cases, systemic signs (fever, weight loss, malnutrition).

Furthermore, patients with IBD may present with signs and symptoms related to the presence of the so-called extraintestinal manifestations (EIMs) of the disease, which are, in fact, other immune-mediated inflammatory disorders (IMIDs). These conditions, while affecting organs outside the gastrointestinal tract, are interconnected through shared inflammatory pathways and underlying pathogenic mechanisms. Overall, approximately 25% of IBD patients are affected by concomitant IMIDs. The most common involve the joints (peripheral and axial spondyloarthropathies), skin (erythema nodosum, pyoderma gangrenosum), eyes (uveitis, episcleritis, iridocyclitis), and hepatobiliary tract (primary sclerosing cholangitis) [3]. However, IMIDs include a wide range of disorders affecting various body systems, such as psoriasis, atopic dermatitis, hidradenitis suppurativa, leukocytoclastic vasculitis, autoimmune hepatitis, autoimmune pancreatitis, type 1 diabetes mellitus, bronchial asthma, and multiple sclerosis. A particular phenotype of IMIDs includes those that develop secondarily to pharmacological treatments for intestinal diseases. These manifestations include the frequent anti-TNF-induced psoriasis and the less common anti-TNF-induced leukocytoclastic vasculitis, drug-induced lupus erythematosus, reactive arthritis/arthralgias, and drug-induced autoimmune hepatitis [4,5]. Several studies suggest that patients with concomitant IMIDs have an increased risk of a more severe disease course [6].

In the case of severe uncontrolled disease, patients with IBD may require hospitalizations and surgical interventions and can experience severely impaired quality of life (QoL) and disability. It is known that patients with IBD have reduced access to education and employment prospects, exclusion from economic and social activities, and challenges in both physical and mental well-being [7]. Moreover, patients with IBD commonly suffer from malnutrition and sarcopenia, which can further worsen clinical outcomes (such as lower therapeutic responses, increased risk of surgery and surgery-related complications), exacerbate disease progression, and compromise growth and development in pediatric patients [8].

Over the past century, there has been a significant evolution in treatment options for IBD: from the absence of specific therapies before the 1930s to the introduction of 5-ASA-based compounds, corticosteroids, and immunomodulators, the so-called “conventional drugs”, followed by the development of advanced classes of medications such as monoclonal antibodies and small molecules [9]. Currently, advanced therapies for IBD available in routine practice include biologics targeting specific inflammatory mediators (anti-TNFα, anti-integrin α4β7, anti-IL12/23, anti-IL23) and small molecules, such as JAK inhibitors and S1P (sphingosine-1-phosphate) modulators (Table 1) [10,11].

The goals of IBD treatment have also evolved in recent years, shifting from simply achieving clinical response to clinical remission, endoscopic response, and finally mucosal healing and beyond.

At present, the targets for medical treatment in clinical practice are defined by the STRIDE consensus, updated in 2021 to its second version [12]. According to STRIDE-II, intermediate targets include the normalization of C-reactive protein (CRP) and a decrease in calprotectin levels. The most important long-term therapeutic targets are clinical remission, restoration of QoL, absence of disability, and endoscopic healing, which has been shown to be associated with significant clinical outcomes, such as steroid-free remission and a decrease in penetrating complications, hospitalization, and surgery [13].

Furthermore, evidence in the literature suggests the possibility of pursuing even more ambitious goals in future clinical practice. Histological healing, defined as the absence of active inflammation in biopsies, has been shown to be associated with favorable prognostic factors in UC, including lower rates of relapse, hospitalization, need for surgery, or medical escalation [14].

On the other hand, in Crohn’s disease, a new therapeutic target that is gaining increasing attention in the literature is transmural healing (TH), which refers to the healing of all layers of the bowel wall. Transmural healing can be assessed through MR enterography or ultrasound and currently does not yet have a standardized definition among clinicians and clinical trials. Despite the variability in the definition, recent studies have reported an association between TH and favorable long-term outcomes, such as lower rates of disease progression over time, fewer relapses, less frequent treatment escalation, and reduced rates of hospitalization and surgery [15].

An even more ambitious target, beyond histological and transmural healing, is complete healing, defined as the absence of both intestinal and extraintestinal inflammation; this could represent the ultimate goal for the treatment of IBD and will likely be investigated in future trials [13].

This evolution towards increasingly ambitious and deeper healing goals, supported by long-term outcome data, has been made possible by advancements in scientific research and the introduction of new medications. However, despite the advent of new drugs, the therapeutic arsenal for the treatment of IBD remains suboptimal and fails to fully meet, in all patients, the diverse clinical challenges and the targets set by the STRIDE consensus and by recent evidence.

## 2. Methods

In this narrative review, we adopted an analytical framework in order to synthesize and assess the current literature evidence. We aimed to describe the chain of logic, as evidence must support possible future clinical outcomes, and to suggest perspectives showcasing future developments in the specific field of the combination of advanced therapies (biologics and small molecules) as a potential therapeutic approach for patients with IBD. To refine the investigation, a comprehensive search strategy was used in order to identify pertinent and relevant literature sources. We used specific Medical Subject Headings (MeSH) terms provided by the National Library of Medicine (NLM). These terms included “ulcerative colitis”, “Crohn’s disease”, inflammatory bowel disease”, “therapy”, “advanced therapy”, “combination therapy”, “biologics”, “anti-TNFalpha”, “anti-integrins”, “anti-cytokines”, “small molecules”, and “JAK-inhibitors”. The primary databases utilized for the literature search included PubMed, Scopus, and Web of Science, with a focus on articles published between January 2021 and November 2024 to capture the most recent developments in the field. We selected articles in English divided into randomized clinical trials, open-label studies, and real-world studies. An additional search was performed on ClinicalTrials.gov in order to identify ongoing clinical trials about CATT in IBD.

## 3. Relevant Sections

### 3.1. Rationale for Combining Therapy in IBD

The gap between therapeutic targets and the efficacy of drug therapy is evident from the very beginning of the therapeutic pipeline, starting with the pivotal trials for new drugs approved for the treatment of IBD. In fact, data from these trials consistently show remission rates below 40%, regardless of the drug class, thereby establishing the so-called “therapeutic ceiling” [16]. One possible explanation, supported by the understanding that the pathophysiology of IBD involves the activation of multiple cell types and cytokine signaling pathways [17], could be that monotherapy (targeting a specific molecular target), may not be the right strategy leading to disease remission in all patients.

Due to these considerations, the field of IBD research is not new to the concept of combining pharmacological therapies. This approach was first explored with the SONIC study, which compared the efficacy of combination therapy with infliximab and azathioprine to each monotherapy in CD patients naïve to immunosuppressors and biologics [18]. Some years later, a similar RCT, the UC SUCCESS trial, was conducted in patients with ulcerative colitis naïve to anti-TNF agents [19]. Both these RCTs demonstrated the superiority of combination therapy in terms of rates of corticosteroid-free clinical remission. Additionally, the SONIC trial showed the superiority of combination therapy in achieving mucosal healing at week 26 (43.9% vs. 30.1% Infliximab monotherapy vs. 16.5% Azathioprine monotherapy). In the UC SUCCESS trial, a significantly greater proportion of patients achieved clinical remission in the combination therapy group at week 16 (29.5% vs. 11.7% with infliximab monotherapy and 13.2% with azathioprine monotherapy).

However, despite the evidence supporting the superiority of combination therapy over monotherapy, the results of the SONIC trial also suggested an increased relative risk of severe adverse events (SAEs) in patients undergoing combination therapy, including serious and opportunistic infections as well as hepatosplenic T-cell lymphoma. In the UC SUCCESS trial, no differences in adverse events (AEs) between treatment groups were noted; however, this was likely due to the short follow-up duration, with adverse events recorded only up to week 8, limiting the ability to detect many severe events.

In clinical practice, these safety concerns generally lead to limiting the duration of patient exposure to combination therapy and carefully selecting patients for whom this therapeutic strategy is appropriate.

The benefit of using two different drugs—a conventional drug and an advanced therapy—with a synergistic effect was also recently observed by Singh et al., in a clinically significant scenario that is rarely the focus of RCTs: acute severe ulcerative colitis (ASUC) [20]. In this study, all patients with ASUC were treated with intravenous steroids and were randomized 1:1 into two groups: one receiving additional therapy with 10 mg of tofacitinib three times daily for 7 days and the other receiving a matching placebo. The rationale for this study was based on evidence that tofacitinib inhibits IL-2 signaling, which has been shown in vitro to alter glucocorticoid receptor expression, thereby contributing to steroid resistance. The results demonstrated that tofacitinib, as add-on therapy to steroids, increases the likelihood of a therapeutic response by day 7 (OR 3.42, 95% CI 1.37–8.48) and reduces the need for medical or surgical rescue interventions both in the short term (7 days) and the long term (90 days) without significant treatment-related side effects.

### 3.2. Combining Advanced Therapies

In recent years, the therapeutic arsenal available for the management of IBD has significantly expanded. After a period of approximately 10 years during which clinicians, in addition to conventional therapy, had access to only drugs within the same therapeutic class and mechanism of action (that is, anti-TNFalplha infliximab, adalimumab, golimumab), the current therapeutic options now include 13 different molecules with six distinct mechanisms of action. Moreover, the biologics and small molecules that have been added to the therapeutic armamentarium are characterized by several advantages, such as subcutaneous or oral administration, which is preferred by patients and less resource-demanding for healthcare systems, reduced immunogenicity compared to infliximab, and favorable safety profiles [21,22]. These positive factors, together with the advent of biosimilars, have led to an increasing use of these agents, particularly in high-income countries, with a corresponding decline in the use of maintenance therapy with immunosuppressants, which are less manageable and have a narrower therapeutic index [23].

However, as highlighted in the previous paragraph, even advanced therapies fail to achieve remission in all patients. This limitation refers not only to efficacy, as demonstrated in pivotal registration trials, but also to effectiveness, as observed by real-world clinical data. A study conducted using data from the Danish National Patient Registry revealed that between 2011 and 2018, prescriptions for biologic therapies increased, although the data on their efficacy were suboptimal. In this study, therapy persistence—assessed as an indicator of both efficacy and tolerability—was found to be low in both the short and long term. Approximately one-third of patients with UC and one-fifth of those with CD discontinued therapy within 12 weeks of initiation (primary failure). In addition, one year after starting therapy, fewer than half of UC and CD patients remained on first-line biologic treatment [24].

The lack of efficacy of advanced therapies can manifest both at the initiation of treatment (primary non-response) and in later stages as a loss of response to treatment (secondary non-response), which, in the case of anti-TNF therapy, may affect up to 30–50% of patients. Several pathogenic hypotheses have been proposed to explain this phenomenon.

The first involves the immunogenicity of these drugs, leading to the formation of neutralizing anti-drug antibodies that reduce treatment efficacy by lowering circulating drug levels [25]. Beyond this, another explanation focuses on changes within the cytokine network underlying the disease in response to anti-TNF therapy. Evidence from the literature suggests that anti-TNF therapy, in some patients, induces upregulation of IL-23 production by CD14+ macrophages, increased IL-23R expression on CD4+ TNFR2+ T lymphocytes, and consequent activation of STAT3 in these cells. This pathway enables these cells to expand and resist anti-TNF-induced apoptosis, thereby perpetuating mucosal inflammation [17,26]. This finding highlights the complexity and dynamic nature of the inflammatory processes underlying IBD, which are not only intricate but also evolve over time in response to pharmacological therapy.

In this complex context, it has been hypothesized that combined therapy with advanced drug classes, such as biologics and small molecules, may represent a solution for several patients with a disease not fully controlled by monotherapy or losing response during follow-up. Despite this concept being relatively new in the field of IBD, it is rapidly evolving. Indeed, it has been defined in different ways according to the evolving therapeutic scenario: initially, it was defined as Dual Biological Therapy, then Dual Targeted Therapy, as well as Advanced Combination Therapy or Advanced Targeted Therapy, and finally, the term Combined Advanced Targeted Therapy (CATT) was used.

The rationale for CATT is therefore to achieve additional therapeutic benefit through different mechanisms such as pharmacokinetic advantages, pharmacodynamic additive or synergistic effects, or simply better chances of responding to a single drug. Ideally, CATT should be effective without increasing the risk of adverse events.

## 4. Current Evidence

Although CATT has become a topic of great interest in the scientific community in recent years, the evidence available in the literature is currently limited, mostly derived from retrospective cohorts and case series, with very few RCTs. In Table 2, we report the evidence coming from studies of the highest scientific quality.

**Figure 1:**
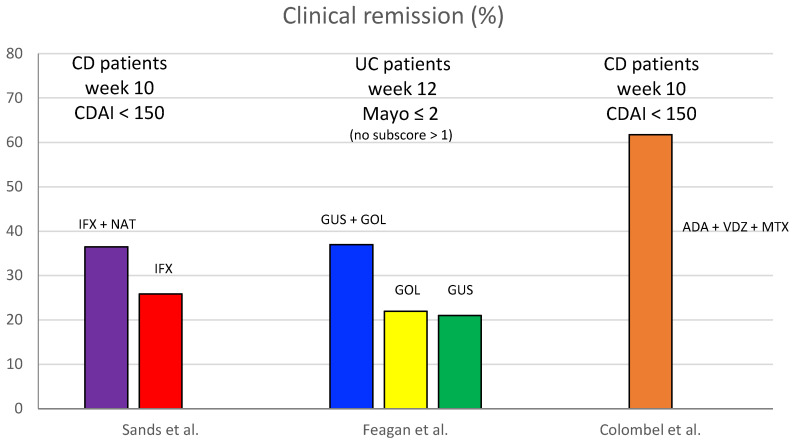
Clinical remission rates from high-quality studies about Combined Advanced Targeted Therapy in IBD [27,28,29]. CDAI = Crohn’s Disease Activity Index; CD = Crohn’s disease, UC = ulcerative colitis, IFX = infliximab, NAT = natalizumab, GUS = guselkumab, GOL = golimumab, ADA = adalimumab, VDZ = vedolizumab, MTX = methotrexate.

In Figure 1 we report the clinical remission rates of the single studies.

### 4.1. Randomized Controlled Trials

The first pioneering study to investigate the effects of CATT in IBD was published by Sands et al. in 2007 and involved 79 patients with Crohn’s disease [27]. This RCT blindly compared monotherapy with infliximab at a standard dose of 5 mg/kg every 8 weeks with combination therapy involving infliximab and natalizumab, a monoclonal antibody directed targeting the α4β1 integrin, at a dose of 300 mg every 4 weeks. The study met the primary safety endpoints, as no statistically significant difference in the incidence of adverse events was observed. However, it failed to demonstrate differences in terms of efficacy between the two therapeutic strategies: while a reduction in the CDAI score was observed in the combination therapy group, it did not prove to be statistically significant.

In 2023, data from a phase 2, proof-of-concept RCT were published, comparing combination therapy with golimumab and guselkumab versus their respective monotherapies in patients with moderate-to-severe ulcerative colitis [28]. Patients, who were naïve to anti-TNF, IL-12/23, or IL-23 antagonists, were randomly assigned (1:1:1) to three treatment arms: golimumab monotherapy (200 mg SC at week 0, 100 mg SC at week 2, then every 4 weeks), guselkumab monotherapy (200 mg IV at weeks 0, 4, and 8, followed by 100 mg SC every 8 weeks), or combination therapy (guselkumab 200 mg IV at weeks 0, 4, and 8, followed by 100 mg SC every 8 weeks, along with golimumab 200 mg SC at week 0, 100 mg SC at weeks 2, 6, and 10). In the combination group, golimumab was discontinued after week 10, and patients continued treatment with guselkumab alone during the maintenance phase. This approach was taken for safety reasons, limiting combination therapy with two biologics to the induction phase only. Placebo administrations (intravenous or subcutaneous) were utilized to maintain blinding. At week 12, the primary endpoint of clinical response (primary endpoint) and remission (secondary major endpoint) rates in the combination therapy group were 83% (59/71) and 37% (26/71), respectively. In the golimumab monotherapy group, response and remission rates were 61% (44/72) and 22% (16/72), respectively, while in the guselkumab monotherapy group, they were 75% (53/71) and 21% (15/71). Notably, statistical significance was observed only in the direct comparison of clinical response at week 12 between combination therapy and golimumab (*p* = 0.0032). In other comparisons, statistical significance was not achieved, though a favorable trend was noted for combination therapy in clinical remission outcomes (*p* = 0.0578 vs. golimumab; *p* = 0.041 vs. guselkumab). In the maintenance phase, at week 38, clinical response and remission rates in the combination therapy group were 69% (49/71) and 44% (31/71), respectively. In the golimumab monotherapy group, response and remission rates were 58% (42/72) and 22% (16/72), respectively, while in the guselkumab monotherapy group, they were 72% (51/71) and 31% (22/71). Regarding adverse events, no differences were observed between the three treatment groups during the observation period.

### 4.2. Open-Label Study

In 2024, a new open-label study was published evaluating the efficacy of combination therapy with vedolizumab, adalimumab, and methotrexate in 55 patients with moderate-to-severe Crohn’s disease diagnosed within the past two years, naïve to any approved or investigational biologic [29]. The study included an initial phase of triple therapy: vedolizumab and adalimumab according to a standard induction schedule (300 mg IV at W0, W2, W6, and then every 8 weeks, and 160 mg SC initially, 80 mg W2, followed by 40 mg every 2 weeks) and methotrexate at a dose of 15 mg orally once a week. Adalimumab and methotrexate were subsequently discontinued at weeks 26 and 34, respectively, leading to the second phase in which patients continued on vedolizumab monotherapy at 300 mg every 8 weeks. The rationale for this combination was to utilize two biologics with distinct mechanisms of action (an anti-TNF-α and a gut-selective α4β7 integrin antagonist) alongside an immunomodulator to potentially reduce biologic-therapy-related immunogenicity. At week 26, 54.5% of patients achieved clinical remission (CDAI score < 150), 56.4% showed endoscopic response (defined as a 50% reduction in SES-CD from baseline), and 34.5% achieved endoscopic remission (defined as SES-CD ≤ 2). Due to the single-arm, open-label design—a limitation of the study—efficacy data were compared post hoc through Bayesian analysis with literature data on monotherapies and a placebo. According to this analysis, the probability of triple therapy achieving endoscopic remission was 99.9% or greater compared to a placebo, 86.3% compared to vedolizumab monotherapy, and 71.4% compared to adalimumab monotherapy. In terms of safety, during triple therapy, 26 therapy-related AEs were reported in 17 patients (30.9%). Of these, seven AEs in six patients were classified as severe, with small bowel obstruction being the most common (two patients, 3.6%). Other SAEs included perianal abscess, fever with lymphadenopathy, acute gastroenteritis, and Crohn’s disease exacerbation.

In conclusion, findings from the EXPLORER study suggest that triple combination therapy has an acceptable safety profile, comparable to safety data of individual monotherapies reported in clinical trials and real-world studies, and may improve endoscopic remission rates in patients with early Crohn’s disease.

### 4.3. Real-World Studies

The first real-world report on the possibility of Combined Advanced Targeted Therapy dates back to 2015, when Hirten and colleagues described the clinical case of a 43-year-old man with severe ileo-colonic Crohn’s disease and multiple prior failures with advanced therapies. In this case, a short course of combination therapy with infliximab and vedolizumab resulted in both clinical and endoscopic improvement. Attempts at monotherapy with either drug led to worsening of the disease: discontinuation of vedolizumab exacerbated intestinal disease, while discontinuation of infliximab triggered worsening of extraintestinal manifestations (EIMs), particularly erythema nodosum [30].

Since then, numerous studies have been published, leading to a meta-analysis by Ahmed and colleagues published in 2022 [31].

This analysis reviewed studies published up to that point that included patients treated with a combination of biologics and/or small molecules, including drugs not FDA-approved for the treatment of IBD, such as etanercept, apremilast, and secukinumab. In total, 30 studies were initially identified, comprising 288 trials of combination involving 279 patients. However, following qualitative analysis, only 10 studies, each including at least 10 patients, were deemed eligible for quantitative analysis. The patients had a mean age of 35 ± 10.1 years and an average disease duration of 12.3 ± 4.6 years, with 76% having CD, 22% UC, and the remainder diagnosed with pouchitis or IBD-unclassified (IBD-U). The primary indication for initiating CATT was refractory intestinal disease in 81% of cases, while 12% started therapy due to concurrent EIMs. The mean treatment duration was 24 weeks, with an average follow-up of 32 weeks, and the median number of prior biologic therapies was two. The most frequently reported combinations were anti-TNF and anti-integrin therapies (48%, vedolizumab or natalizumab), followed by anti-integrin combined with ustekinumab (19%). Tofacitinib was used in combination therapy in 20% of cases, with vedolizumab accounting for the majority of these combinations. Pooled results from this meta-analysis revealed clinical response and remission rates of 69.2% (95% CI 51.5–84.4%) and 58.8% (95% CI 42–74.5%), respectively, with endoscopic response and remission rates of 42.9% (95% CI 6.9–59.6%) and 34.3% (95% CI 23.5–46.1%), respectively. Among patients with active EIMs at baseline, the pooled response rate to therapy was 49.9% (95% CI 14.2–85.7%), although significant heterogeneity across studies was noted for this outcome. Regarding safety, the pooled rates of adverse events (AEs) and serious adverse events (SAEs) were 31.4% (95% CI 12.9–53.7%) and 6.5% (95% CI 2.1–13.1%), respectively.

Another meta-analysis, conducted by Alayo and colleagues, was published in 2022 [32]. Unlike the previous study, this analysis focused exclusively on combinations of drugs approved by the FDA for the treatment of IBD. Additionally, pooled data were presented for each specific drug combination. This meta-analysis included 13 studies comprising 266 patients and 271 treatment trials across seven different types of combination therapies. Among the patients, 70.7% had a diagnosis of CD, 28.2% had UC, and 1.1% had IBD-U. The median number of prior advanced therapies ranged from 0 to 4, with median follow-up durations ranging from 16 to 68 weeks. The two most frequently reported combinations were vedolizumab + anti-TNF (56 cases) and vedolizumab + tofacitinib (57 cases). The pooled results for clinical remission varied from 40.4% for the ustekinumab + tofacitinib combination to a maximum of 80% for ustekinumab + anti-TNF. Vedolizumab + anti-TNF and vedolizumab + tofacitinib yielded pooled remission rates of 55.1% and 47.8%, respectively. Endoscopic or ultrasound remission rates ranged from 18% for vedolizumab + anti-TNF to 37.4% for ustekinumab + tofacitinib, with vedolizumab + tofacitinib achieving a pooled rate of 24.6%. Regarding safety, pooled rates of adverse events (AEs) ranged from 6.7% for anti-TNF + tofacitinib to 92.3% for anti-TNF + natalizumab. Serious adverse events (SAEs) ranged from 0% for combinations such as anti-TNF + natalizumab, anti-TNF + ustekinumab, anti-TNF + tofacitinib, and ustekinumab + tofacitinib up to a maximum of 12.3% for ustekinumab + vedolizumab. Notably, for all combination therapies, approximately 75% of the SAEs consisted of infectious events.

Both meta-analyses conclude that CATT demonstrated acceptable safety profiles, similar to those reported for individual monotherapies, with no reports of new adverse events. Furthermore, the results suggest the effectiveness of CATT in the treatment of IBD.

### 4.4. Ongoing Trials

Following the promising results from the VEGA study, two trials are still ongoing to evaluate the efficacy of combination therapy with golimumab and guselkumab in patients with Crohn’s disease (DUET-CD, NCT05242471 on ClinicalTrials.gov accessed on 15 December 2024) and in those with moderate-to-severe ulcerative colitis (DUET-UC, NCT05242484) [33]. Both studies are phase 2, randomized, double-blind trials with six treatment arms: placebo, guselkumab monotherapy, golimumab monotherapy, and three combination therapy arms (JNJ-78934804, a combination of guselkumab and golimumab) at different dosages (high, medium, low). The primary outcomes are clinical remission at week 48 for both studies and endoscopic response at week 48 for DUET-CD. According to the latest updates available at the time of this article’s publication, initial results from both studies are expected starting in May 2025.

At the time of publication of this article, three clinical trials on CATT with vedolizumab are currently registered on ClinicalTrials.gov [33]. All three are still in the early stages, with two in the recruitment phase. One of these trials is an open-label, single-arm study conducted in patients with ulcerative colitis, aimed at evaluating combination therapy with vedolizumab and tofacitinib (NCT06095128). The other two studies focus on Crohn’s disease: one is designed to compare vedolizumab + adalimumab with vedolizumab + ustekinumab (NCT06045754), while the other compares vedolizumab monotherapy with vedolizumab + upadacitinib (VICTRIVA, NCT06227910). All three studies consist of two phases: an initial phase of varying duration, during which patients receive the combination therapy, followed by a maintenance phase in which only vedolizumab therapy is continued. The estimated primary completion for all three studies is no earlier than 2027.

## 5. Discussion

The management of IBD has become a key area of research, with a focus on developing new therapeutic options for clinical practice. Over the past five years alone, the European Medicines Agency (EMA) has approved several drugs for IBD, with additional treatments under investigation and expected to be available in the coming years.

However, despite the increasing number of available drugs, achieving the goals outlined by the STRIDE II consensus and recent evidence in all patients remains challenging. This treatment gap not only contributes to persistent clinical disease activity in many patients but, from a greater perspective, results in high direct and indirect costs associated with IBD, along with a significant demand for healthcare resources.

Considering these aspects and based on IBD’s complex pathophysiology involving multiple cytokine pathways, the scientific community has begun to explore the hypothesis that combining two distinct advanced therapies may offer enhanced efficacy. Combination therapies, in fact, are already widely used in other fields of medicine to treat complex conditions such as HIV, tuberculosis, cancer, and cardiovascular diseases. Furthermore, combinations of conventional drugs and advanced targeted therapies have already proven beneficial in the treatment of IBD and are, in some cases, part of routine clinical practice.

One of the most important limitations in pursuing combination therapy with immunomodulant agents is represented by the fear of side effects, mainly infectious or neoplastic. In IBD, the research in this field has been slowed down by the safety concerns emerging from studies using a combination of advanced therapies in rheumatology. Indeed, given the scarcity of data available on CATT for the treatment of IBD, previously, rheumatology data were often considered a useful reference for IBD treatment strategies, owing to the extensive use of combination therapies in rheumatology [34]. However, despite belonging to the group of IMIDs, rheumatologic diseases and IBD exhibit substantial differences. These differences arise from the intrinsic characteristics of the diseases and the affected populations, with IBD patients typically being younger and having fewer comorbidities, and the therapeutic agents employed, which often differ in terms of mechanism of action and safety profiles. Therefore, evidence regarding the treatment of one disease, in terms of both efficacy and rate of adverse events, should not be extended to the other. In this direction, it is helpful to report the results of a recent meta-analysis conducted by Solitano et al. [35]. In this meta-analysis, phase 2 and 3 RCTs on combination therapy with biologics and/or small molecules were included, encompassing both IBD (two trials) and rheumatologic IMIDs (eight trials: seven in rheumatoid arthritis and one in lupus nephritis). The results of the study suggest, despite with low certainty of evidence, that CATT in rheumatoid arthritis does not provide additional benefits and is associated with an increased risk of adverse events. Conversely, in IBD, CATT appears to offer benefits compared to monotherapy without an increased risk of adverse events.

This report is consistent with what emerged in the previous sections of this article. In IBD, CATT has shown promising efficacy and effective data. Additionally, this approach appears safe and seems to lack severe adverse events, although current data are limited and mainly derived from short-term studies that may not capture long-term outcomes like malignancies or mortality.

Combined Advanced Targeted Therapy, in addition to representing a novel resource for the treatment of “difficult-to-treat” luminal IBD, may also serve as a valuable therapeutic option for patients with extraintestinal manifestations and immune-mediated inflammatory diseases (IMIDs). In these patients, immune system dysregulation extends beyond the gastrointestinal tract, representing part of a systemic process, therefore suggesting the need for a more holistic approach that can simultaneously address different inflammatory pathways. Furthermore, the presence of concomitant IMIDs has been associated with a more aggressive course of intestinal disease, further supporting the consideration of more aggressive therapeutic strategies [36]. In fact, in clinical practice, when patients present with overlapping IMIDs, combined advanced therapies are already being prescribed by different specialists, such as gastroenterologists and rheumatologists, to simultaneously address both the luminal disease and the extraintestinal manifestations [37].

Nonetheless, several questions and challenges remain for the future.

First of all, the applicability of this approach in everyday clinical practice is still limited by the low level of evidence. In particular, the available literature lacks recent and reliable RCTs. The most recent publication about CATT is an open-label single-arm study [29], which lacks a comparison group (e.g., placebo or monotherapy) and therefore limits the ability to definitively conclude the superiority of that therapy; additionally, it has been conducted in patients with early Crohn’s disease naïve to advanced therapies, who, as documented in the literature, show higher treatment response rates compared to those with longer disease duration or prior failures to biological therapies. Moreover, real-world data mainly come from retrospective observational studies, using various therapeutic combinations in small sample sizes, for different indications and considering different clinical outcomes. Further limitations of these findings include the short follow-up period, which makes it challenging to capture major outcomes such as surgery, death, or malignancies.

Additionally, the drug combinations tested so far have been chosen based on biological plausibility, as no evidence currently guides the optimal combinations. The challenge in treating IBD, unlike in oncology—where therapy may be tailored to the immunohistochemical phenotype of the tumor—is that treatment decisions are influenced by various factors depending on the different features of the disease (e.g., phenotype, age at diagnosis, previous therapeutic failures, previous surgery) and of the patient (e.g., age, comorbidities, preferences/adherence, nutritional status, frailty), as well of the medications (e.g., availability, route of administration, cost, safety profile, rapidity of action). In fact, in IBD, we lack markers or predictive factors to profile patients and assist clinicians in identifying the drug class with the highest likelihood of response. A key future challenge will be to discover markers or predictive factors that can guide clinicians in selecting the therapy most likely to be effective, thereby avoiding “blind trial-and-error” approaches.

Currently, the choice of combination therapy can be tailored based on specific disease characteristics and the presence of associated IMIDs. For instance, in patients with perianal disease, a combination therapy involving infliximab may be particularly beneficial. In cases of concomitant spondyloarthropathies, therapies including anti-TNF agents or upadacitinib are recommended. Similarly, in the presence of atopic dermatitis or psoriasis, combinations incorporating upadacitinib or anti-IL-23 therapies, respectively, would be appropriate [38].

Furthermore, there is uncertainty about the optimal therapeutic protocol to adopt and the ideal duration for combination therapy.

Regarding the induction phase, potential treatment strategies include “co-induction” or “simultaneous induction”, where the two drugs comprising the combination advanced targeted therapy (CATT) are initiated simultaneously. This approach may be particularly beneficial for patients with predictive factors of greater disease severity, such as the need for hospitalization or surgery. Examples of such situations include early onset, extensive disease, the presence of deep ulcers, perianal disease, and a penetrating phenotype in Crohn’s disease. In ulcerative colitis, high-risk factors include early onset, pancolonic involvement, and a history of frequent flares requiring steroids or hospitalization [39].

Alternatively, the second drug could be added later, after the initiation of the first and upon evidence of monotherapy insufficiency in achieving therapeutic goals (“add-on therapy”). Another option is to complete the induction phase with one drug and subsequently switch to another for maintenance to sustain its effectiveness [40].

For maintenance therapy, since the early stage is critical for reducing organ damage and disease progression [41], it is plausible that the combined effect of two drugs could be limited to induction, followed by maintenance with a single agent offering a better safety profile (e.g., vedolizumab, ustekinumab, anti-IL-23) (“step-down approach”).

Alternatively, maintenance strategies might involve continuous CATT, utilizing various potential combinations of advanced therapies. For instance, a combination of anti-TNF and anti-IL-23 agents could be considered, given their complementary safety profiles and the emerging evidence on IL-23-dependent mechanisms contributing to a loss of response to anti-TNF therapies. Another potential option is “intermittent reinduction”, where a primary therapy is maintained continuously and a second agent is introduced periodically in short, cyclic courses to either sustain disease control or restore remission during flares [40].

This latter approach is particularly intriguing as it is more dynamic and tailored to disease phenotype, which can vary. While some patients exhibit a chronically active course, others may experience an intermittent pattern, characterized by mild baseline activity interspersed with severe flare-ups. A differentiated management strategy based on disease course, rather than adhering to a “chronic maintenance therapy” model, could be beneficial for these patients. This strategy is particularly feasible with the advent of rapid-acting and less immunogenic drugs, such as JAK inhibitors, which are well suited to this type of approach. On the other hand, this therapeutic policy requires continuous patient monitoring to promptly identify flares or even signals indicating an increased short-term risk of flare, allowing for timely therapeutic escalation. To ensure both patient adherence and cost-effectiveness, monitoring should prioritize non-invasive methods, such as biochemical markers (e.g., C-reactive protein, fecal calprotectin) and ultrasound.

Nonetheless, one of the major challenges in implementing CATT in IBD management may be represented by the associated costs, and, consequently, the sustainability of this therapeutic strategy for healthcare systems. However, the introduction of more biosimilars and the time-limited use of combination therapy in certain regimens, either at the induction phase or intermittently, may further support the approach of CATT by lowering costs and ensuring economic sustainability.

## 6. Conclusions

**Figure 2:**
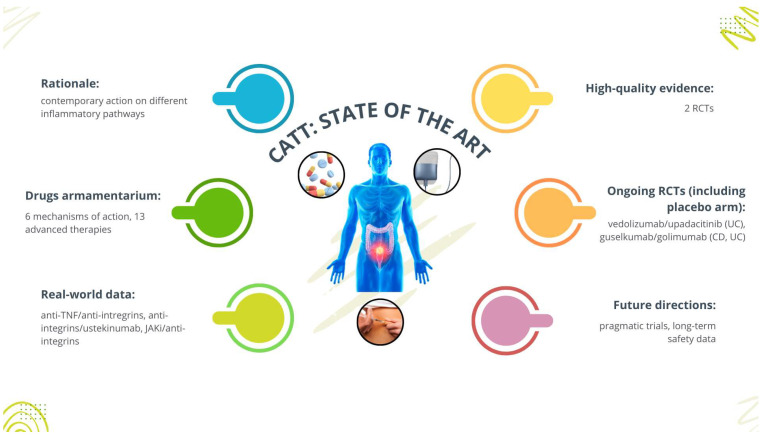
Combined Advanced Targeted Therapy (CATT) in IBD.

The current situation with CATT in IND is summarized in Figure 2.

In IBD, initial data on CATT are promising, and this approach will likely become increasingly prominent; however, more longitudinal studies, particularly RCTs, are necessary. Ideally, these trials should compare combination therapies—potentially with different treatment duration arms—against the standard of care (monotherapy with a biologic or small molecule), rather than a placebo. Moreover, the study population should closely reflect the patient profile encountered in clinical practice, including, for instance, those who have failed multiple lines of treatment and elderly patients. This latter group of patients represents a condition that physicians managing IBD will encounter more and more often in the near future. The management of patients with elderly-onset IBD and of elderly patients with IBD diagnosed at a younger age deserves specific therapeutic considerations and decisions. In particular, common comorbidities, malnutrition, increased risk of malignancies, and increased disposition to infections (mainly serious and opportunistic) can render elderly patients more vulnerable to complications of immunosuppression. Moreover, polypharmacy from existing comorbidities is common in elderly patients, and the potential for drug interactions must be considered as well as the impact on adherence.

Finally, long-term follow-up data about Combined Advanced Targeted Therapy are essential to identify adverse events that may not be immediately evident.

As more evidence becomes available, it will progressively be incorporated into routine clinical practice.

At present, it should be noted that CATT is not an approved strategy and should therefore be considered only for selected patients, preferably within specialized centers capable of providing an interdisciplinary approach. For an effective collegial discussion, a detailed assessment of the patient’s clinical history, including information such as previous failures, responses, and adverse events, is mandatory. Furthermore, clinical management should focus on a comprehensive evaluation of the patient, encompassing not only the pharmacological aspects but also the assessment of other aspects such as age, nutritional status, frailty, comorbidities, and psycho-social status. Addressing factors like malnutrition and sarcopenia is critical to mitigating their negative impact on disease progression and promoting an overall improvement in the patient’s quality of life [8].

The use of combination therapy should be limited to a short period, with strict monitoring of treatment response and safety to minimize potential adverse effects. Notably, multidisciplinary discussions should provide patients with a comprehensive and transparent disclosure of the risks and benefits associated with the proposed therapeutic strategies. Patient involvement in the care pathway is essential, ensuring clear communication not only about potential drug side effects but also about the consequences of untreated IBD.

**Figure 3:**
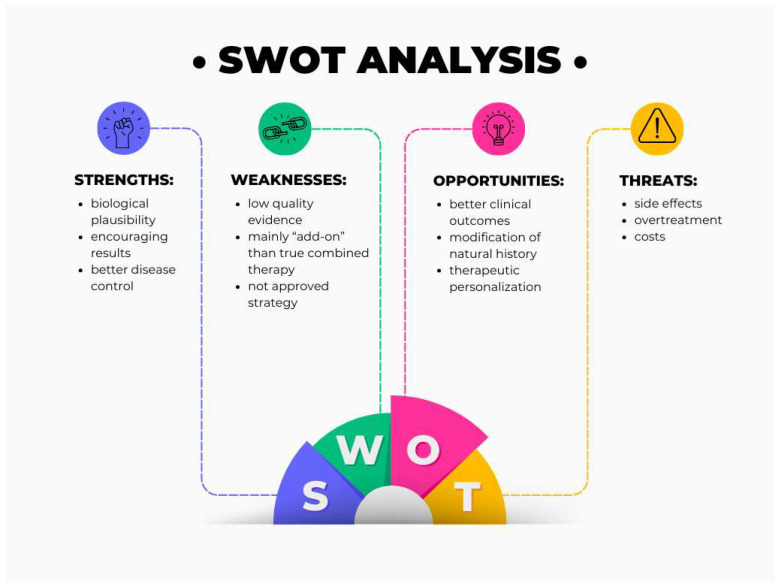
A SWOT analysis for Combined Advanced Targeted Therapy in IBD.

A SWOT analysis of CATT in IBD is provided in Figure 3.

## Figures and Tables

**Table 1:** Target of therapies currently available for IBD (in bold, advanced therapies).

Target	Drug
Untargeted anti-inflammation	Salicylates, steroids, immunosuppressants
Cytokines promoting inflammation and/or inducing differentiation or maintenance of inflammatory immune cells	**TNF antagonists** **IL-12/IL-23 antagonists** **IL-23 antagonists**
Multiple cytokine signaling pathways	** JAK inhibitors **
Modulation of lymphocyte trafficking	** Anti-integrins, S1PR modulators **

TNF = Tumor Necrosis Factor; IL-12 = Interleukin 12; IL-23 = Interleukin 23; JAK = Janus kinase; S1PR = sphingosine-1-phosphate receptor.

**Table 2:** Evidence from high-quality studies about Combined Advanced Targeted Therapy in IBD.

Author (Year)	Study Design	Population	Type of Combination	Outcome
Sands et al. (2007) [27]	RCT	CD (79)	IFX + Natalizumab vs. IFX	Clinical remission W2 (15.4%), W6 (23.1%), and W10 (36.5%) vs. W2 (7.4%), W6 (25.9%), and W10 (25.9%)
Feagan et al. (2023) [28]	RCT	UC (214)	Group A: GOL + GUS until W12, then only GUS vs. Group B: GOL vs. Group C: GUS	W12 clinical response and remission: Group A 83% and 37%, B 61% and 22%, C 75% and 21% W12 endoscopic response and remission: Group A 49% and 18%, B 25% and 10%, C 72% and 8% W38 clinical response and remission: Group A 69% and 44%, B 58% and 22%, C 72% and 31% W38 endoscopic response and remission: Group A 49% and 25%, B 22% and 7%, C 32% and15%
Colombel et al. (2024) [29]	Single-arm open-label	CD (55)	Initial phase: VDZ + ADA + MTX, then discontinuation of ADA (W26) and MTX (W34). Maintenance: VDZ	W10 and W26 clinical remission: 61.8% and 54.5% W10 and W26 clinical response: 47.3% and 43.6% W26 endoscopic response 56.4% W26 endoscopic remission 34.5%

RCT = randomized controlled trial, CD = Crohn’s disease, UC = ulcerative colitis, IFX = infliximab, GOL = golimumab, GUS = guselkumab, VDZ = vedolizumab, ADA = adalimumab, MTX = methotrexate.

## Data Availability

No new data were created.
